# Plasma proteome-wide Mendelian randomization reveals multi-ancestry drug targets for gastric cancer

**DOI:** 10.3389/fonc.2026.1821512

**Published:** 2026-05-01

**Authors:** Peng Zhi, Yan Cui, Guanghui Xue, Lingyu Qiao, Jie Geng, Zhengyao Chang, Yinmei Xu, Juanjuan Yan, Yingli Wang, Chenghui Zhao

**Affiliations:** 1 Experimental management center, Shanxi University of Chinese Medicine, Jinzhong, Shanxi, China; 2 Shanxi Research Center for Chinese Medicine Development, Jinzhong, Shanxi, China; 3 Department of Ophthalmology , Affiliated Hospital of Shanxi University of Chinese Medicine, Taiyuan, Shanxi, China; 4 Basic Medicine College, Shanxi Medical University, Taiyuan, Shanxi, China; 5 Department of General Surgery, The First Medical Center, Chinese PLA General Hospital, Beijing, China; 6 Department of Respiratory and Risk severe case, The third Medical Center, Chinese PLA General Hospital, Beijing, China; 7 School of Public Health and Health Management, Shanxi University of Medicine, Fenyang, Shanxi, China; 8 School of Health Service and Management, Shanxi University of Chinese Medicine, Jinzhong, Shanxi, China; 9 Medical Innovation Research Division, Chinese PLA General Hospital, Beijing, China

**Keywords:** drug targets, gastric cancer, Mendelian randomization, molecular docking, multi-ancestry, single-cell RNA sequencing

## Abstract

**Background:**

Gastric cancer (GC) exhibits marked epidemiological differences between European (EUR) and East Asian (EAS) populations, with significantly higher incidence rates in EAS. Circulating proteins represent promising drug targets; however, most proteomic studies have focused primarily on EUR ancestry, leaving EAS-specific targets largely underexplored. This study aims to identify ancestry-specific plasma protein targets for GC using Mendelian randomization (MR).

**Methods:**

We employed Mendelian randomization (MR) and colocalization approaches to assess the putative causal effects of plasma proteins on GC risk across diverse populations. Specifically, we examined proteins in EUR and EAS populations. Subsequent single-cell RNA sequencing and molecular docking were conducted to identify cellular expression patterns and potential therapeutic compounds. Finally, experiments were conducted to verify the newly discovered drug target.

**Results:**

Our analyses revealed four significant protein targets in the EUR population (SLURP1, ANGPTL3, NME4, ANXA10) and nine in the EAS population (ICAM5, SMOC1, PSCA, SATB1, SCRG1, PTPRB, ISLR2, NCR3LG1, SELE) associated with GC susceptibility. These drug targets are mainly expressed in epithelial cells, and Pit mucous cells in gastric tumor tissue. Experimental validation indicated significant downregulation of NME4 and upregulation of ICAM5 in GC cell lines and tissue samples, suggesting potential tumor-suppressive and oncogenic functions, respectively. Moreover, molecular docking identified seven repurposable drugs, with digoxin demonstrating cross-ancestry therapeutic potential.

**Conclusion:**

Our findings delineate 13 ancestry-specific protein targets, including five novel candidates, thereby enhancing our understanding of GC pathogenesis. This study underscores the importance of ancestral diversity in informing drug development strategies for GC. Future research should focus on validating these targets and exploring their functional mechanisms further to translate these findings into clinical applications.

## 1 Introduction

The latest GLOBOCAN estimates indicate that gastric cancer (GC) is the fifth most prevalent malignancy globally, with 968000 new cases reported in 2022 (1). Notably, the highest incidence rates of GC are observed in East Asia (EAS), with a rate of 32.7 per 100,000 individuals, followed by Eastern Europe (EUR) at 23.9 per 100,000 (2).The standard treatment paradigm for GC encompasses a multimodal approach, including surgery, chemotherapy, radiotherapy, and targeted molecular therapies (3). GC is predominantly diagnosed at advanced stages, a clinical characteristic strongly associated with unfavorable prognostic outcomes (4). Integrating the diagnostic challenge and the need for novel biomarkers, biomarkers can target various aspects of GC carcinogenesis, and their identification as therapeutic targets could significantly enhance treatment efficacy and improve clinical outcomes in GC patients (5).

Circulating proteins, which are secreted or released by cancer cells or cancer-associated cells, have been demonstrated to play a crucial role in various biological processes and are increasingly acknowledged for their potential contribution to GC development and progression (6). These proteins constitute a significant reservoir for cancer druggable targets. While existing studies endorse the utility of circulating proteins as druggable targets (7), they are typically confined to a limited array of proteins, and their effectiveness may be compromised by factors such as confounding influences and issues of reverse causality (8). Recent advances in large-scale proteomics have significantly expanded our understanding of protein quantitative trait loci (pQTLs), with comprehensive studies identifying over 7847 proteins from 89778 participants (9). These extensive datasets provide an unprecedented opportunity to systematically investigate the causal relationships between plasma proteins and cancer risk through Mendelian randomization (MR) approaches. The MR framework has been successfully implemented in identifying cancer drug targets across multiple malignancies, including colorectal cancer (10), lung cancer (11), and various other cancer types (12). Notably, a recent study by Jin et al. identified twelve plasma proteins as promising therapeutic targets for GC, further validating the potential of this approach (13).

However, most of the published proteome-wide genome-wide association studies and association analysis studies have mainly focused on the European population (13). It is also worth noting that GC has a unique epidemiological pattern and molecular pathogenesis. Due to the difference in Helicobacter pylori infection rates between the EAS and the EUR, and the stronger virulence of the dominant strains in the EAS population, the association with the risk of GC is more closely related (14). Additionally, dietary habits such as high-salt diet and consumption of pickled foods are more prevalent in the EAS population (15). This leads to a significantly higher incidence and mortality rate in the EAS population compared to the EUR population. These population-specific differences highlight the urgent need for conducting comprehensive multi-population studies to identify population-specific drug targets.

In the present study, we conducted a proteome-wide MR analysis by integrating plasma proteome data and multi-source GC genome data from individuals of EUR and EAS ancestries. The primary objective of this study was to identify potential drug targets for GC. Furthermore, we conducted colocalization analysis, SMR analysis, and HEIDI test to ascertain drug targets of varying levels of evidence. Subsequently, single-cell RNA sequencing (scRNA-seq) was deployed to delineate the distribution of these biomarkers across diverse stages and cellular lineages. Additionally, we predicted drug targets and conducted molecular docking to identify potential drugs that may have therapeutic effects. At the same time, experiments were used to verify the newly discovered targets.

## 2 Materials and methods

### 2.1 Outcome data source

The current study procured GWAS data from two ancestries, EUR and EAS. For the EUR ancestry, we leveraged data from FinnGen R11 (16) (1,741 cases, 345,118 controls), which offers homogeneous Finnish population genetics to minimize stratification bias; the UK Biobank (17) (994 cases, 457,179 controls), whose extensive phenotypic coverage facilitates pleiotropy analysis; and the study by Jiang et al. (18) (192 cases, 456,156 controls), a GC-specific dataset that enhances statistical power when combined with the others. For the EAS ancestry, we included two GWAS summary datasets from the IEU Open GWAS project (19) (7,921 cases, 159,201 controls) and the JEWEL study (20) (6,563 cases, 195,745 controls). These represent the largest publicly available EAS GC GWAS datasets; the JEWEL study specifically captures the high-incidence Japanese population, and their combined use improves result robustness. **Supplementary Table 1** delineates the specifics of the data sources employed in this study.

### 2.2 Exposure data source

**Figure 1:**
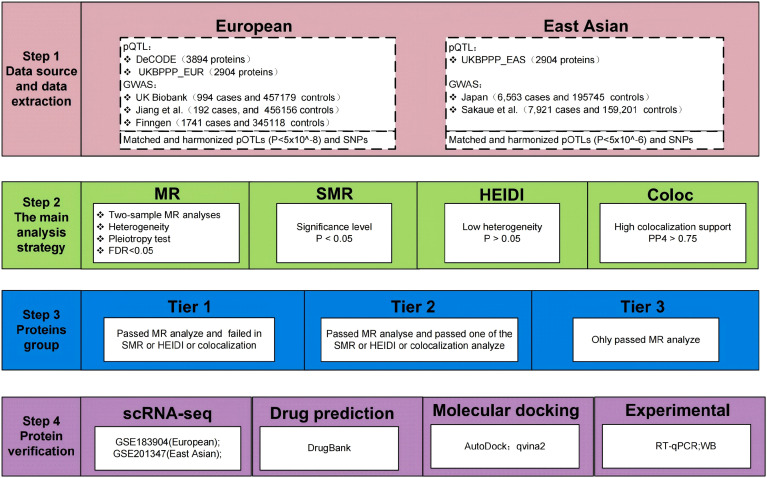
The workflow of the study design. MR, Mendelian Randomization; FDR, false discovery rate; SMR, Summary data-based MR.


The data and analysis process of this study are illustrated in **Figure 1**. We included pQTL data from diverse ancestries. For the EUR, we utilised the DeCODE (21) (https://www.decode.com/summarydata/) and the UK Biobank Pharma Proteomics Project (UKBPPP, https://registry.opendata.aws/ukbppp/) (22) as exposure data. For the EAS, we used UKBPPP data as exposure data. The DeCODE database contained data on 4,907 proteins, while UKBPPP provided data on 2,940 proteins. The instrumental variables (IVs) employed in this study encompass both cis pQTL and trans pQTL. The definition of cis-pQTL is an SNP located within 1Mb of the transcription start site of a protein-coding gene, while trans-pQTL refers to SNPs located outside this region (10). The selection criteria for IVs are as follows: 1) For the EUR, we screened for significant SNPs with P *<* 5 × 10^−8^. A notable difference in pQTL discovery sample size exists between ancestries. For the EUR population, large-scale datasets such as the UK Biobank Pharma Proteomics Project (UKBPPP) provide approximately 90,000 individuals, whereas available EAS pQTL data comprise only several thousand individuals. Consequently, applying the standard genome-wide significance threshold (*P <* 5 × 10^−8^) to the EAS population yielded insufficient cis-pQTL instrumental variables for most proteins. To enable MR analysis in this ancestry, a relaxed threshold (*P <* 5 × 10^−6^) was adopted, a strategy that has been widely used in published MR studies in East Asian populations (23). 2) Linkage disequilibrium was utilised to identify independent associations of pQTLs for each protein (r^2^*<* 0.001). 3) The strength of genetic instruments was estimated using the following formula (24):

$$R^{2}=2\times \text{EAF}\times (1-\text{EAF})\times \beta^{2}$$

$$F=R^{2}\times \frac{N-2}{1-R^{2}}$$

where R^2^ represents the proportion of variation explained by SNPs at the protein level. The higher the R^2^ value, the better the explanatory power. The F-statistic was used to measure the strength of the association between pQTL and GC. We selected IVs with F-statistic *>* 10. As a result, the DeCODE database included 3,894 proteins, while the UKBPPP included 2,904 proteins(**Supplementary Tables S2**–**S4**).

### 2.3 Two-sample MR analysis

For ancestries with multiple outcome GWAS datasets, we first performed separate two-sample MR analyses for each GWAS dataset against the pQTL data, including inverse-variance weighted (IVW), simple mode, weighted mode, weighted median, MR-Egger, and the Wald ratio. For analyses utilizing multiple single nucleotide polymorphisms (SNPs) as instrumental variables, the IVW method was applied to estimate causal effects through weighted linear regression with the intercept constrained to zero. The Wald ratio was used when only a single SNP was available. To control for false discovery rate due to multiple testing, the Benjamini-Hochberg procedure was applied, and plasma proteins with a corrected Pvalue below 0.05 were considered statistically significant. Heterogeneity across instrumental variables was evaluated using Cochran’s Q test, where a P-value greater than 0.05 indicated no significant heterogeneity. Directional horizontal pleiotropy was assessed using the MR-Egger intercept test, with a P-value greater than 0.05 suggesting no evidence of pleiotropy. All statistical analyses were conducted using R software (version 4.3.0) with the “TwoSampleMR” package.

### 2.4 SMR analysis

The software SMR v1.3.1 was employed to perform SMR analysis and HEIDI tests on proteins that were found to be significant in the MR analysis (25). For the SMR analysis, a threshold of statistical significance was set at *P <* 0.05. The HEIDI test was utilized to identify pleiotropy, and the p-value exceeding 0.05 in the HEIDI test suggested that the relationship between the exposure and outcome was not attributable to linkage disequilibrium.

### 2.5 Bayesian colocalization analysis

We executed a Bayesian colocalisation analysis employing the ‘coloc R’ package (26). This method harnesses GC GWAS and pQTL data to discern the colocalisation locus. The colocalisation analysis was structured around five hypotheses: 1) the absence of association with either protein or GC within the genomic locus (H0); 2) the presence of an association variant exclusive to the protein (H1); 3) the presence of an association variant exclusive to GC (H2); 4) the existence of two distinct association variants for both protein and GC (H3); 5) the concurrence of association for both traits, suggesting a common causal genetic variant (H4). A posterior probability of H4 (PPH4) surpassing 0.75 is interpreted as providing robust evidence for the colocalisation of these two signals.

### 2.6 Analysis of single-cell RNA sequencing (scRNA-seq) in GC

ScRNA-seq data from *in situ* cancer tissues of GC patients were obtained from the Gene Expression Omnibus (GEO) database, using GSE183904 (27) for EUR and GSE201347 (28) for EAS. Using the FindClusters function with a resolution parameter of 0.2. We employed the ‘Seurat’ package (29) to filter cells based on the criteria 200 *< nFeature RNA <* 6000 and *percent.mt <* 20. Following logarithmic normalization of the sequencing data, we identified 2000 highly variable genes using the FindVariableFeatures function. We then scaled and normalized the data for these highly variable genes and performed principal component analysis (PCA) to reduce the data’s dimensionality. To evaluate the explanatory power of each principal component, an ElbowPlot was utilized to determine the optimal number of principal components to retain. Subsequently, UMAP dimension reduction was executed based on the outcomes of the first 20 principal components. Neighboring cells were identified using the FindNeighbors function, cluster analysis was conducted at a resolution of 0.2 with the FindClusters function, and the DimPlot function was applied to generate a cell clustering diagram. The FindAllMarkers function was used to detect differentially expressed genes (DEGs) across various cell clusters, with thresholds of min.pct = 0.5, log_2_FC = 1, and *adjp <* 0.05. The identification of cell types was conducted through a comprehensive review of scientific literature from PubMed and the cellmarker database. Then, we verified the expression patterns of genes with a strong association to GC development obtained from MR analysis in the GC microenvironment, including strongly related genes from EUR and EAS. The FeaturePlot function describes the expression distribution of genes in different cell clusters. The DotPlot function was then utilised to demonstrate the specific expression of proteins in different cell clusters.

### 2.7 Drug target identification and molecular docking

We sourced potential drug target proteins from DrugBank (30) and DGIdb (31) to ascertain their corresponding target drugs. For proteins lacking known target drugs, we initiated a structure-based drug screening protocol. Three-dimensional structural models of these proteins were obtained from UniProt, RCSB PDB, and, where necessary, the AlphaFold database. The selection criteria prioritized co-crystal structures solved by X-ray diffraction with a resolution better than 2.50A.˚ All FDA-approved drugs were extracted from the Guide to Pharmacology database (version 2024.3), and their three-dimensional structures were downloaded from PubChem (32). A total of 1,622 small-molecule drugs were used for virtual screening. Prior to molecular docking, all protein and ligand structures were pre-processed using AutoDock Tools (33), including removal of water molecules, addition of hydrogen atoms, and assignment of atomic charges. High-throughput semi-flexible docking was performed using qvina2 (34), which conducts a global search to evaluate binding affinities for each protein–small-molecule pair. Only poses with a predicted binding energy below 7.0kcal/mol were retained for further analysis. For each target protein, the top five drug candidates were then selected based on their docking scores (binding energies). Finally, the protein–drug interaction network was visualized using the ggplot2 function in R, and representative binding modes were displayed in three dimensions using PyMOL to reveal key residues and ligand interaction details.

### 2.8 Experimental validation of candidate targets

Total RNA was extracted from GC cell lines (AGS, HGC-27, MKN-45, SGC-7901) and normal GES-1 cells using Trizol reagent (Tiangen, China). RNA purity (A260/A280 = 1.8-2.0) and integrity (intact 28S/18S rRNA bands) were confirmed by spectrophotometry and agarose gel electrophoresis, respectively. Reverse transcription was performed using the RevertAid First Strand cDNA Synthesis Kit (AG, China) with 200 ng of total RNA. RT-qPCR was conducted on a CFX Connect Real-Time PCR Detection System (Bio-Rad, USA) using PerfectStart Green qPCR SuperMix (TransGen, China), with GAPDH as the internal control. The thermocycling conditions were as follows: initial denaturation at 95 °C for 5 min; 40 cycles of denaturation at 95 °C for 10 s, annealing at 58 °C for 20 s, and extension at 72 °C for 20 s; followed by melting curve analysis from 60 °C to 95 °C. The annealing temperature of 58 °C was optimized based on the melting temperatures of the primers (55-60 °C), and the extension time of 20 s was sufficient for amplicons ranging from 76 to 197 bp. Relative gene expression was calculated using the 2Ct method. All reactions were performed in triplicate. Data were analyzed using Student’s t-test or one-way ANOVA, with P *<* 0.05 considered statistically significant.

Total protein was extracted from GC cell lines (AGS, HGC-27, MKN-45, SGC-7901) and normal GES-1 cells using RIPA lysis buffer (Servicebio, China) containing protease and phosphatase inhibitors. Protein concentrations were determined using the BCA assay (Servicebio). Equal amounts of protein (30 g) were separated by 10% SDS-PAGE and transferred onto PVDF membranes (Millipore, USA). After blocking with 5% non-fat milk, membranes were incubated overnight at 4 °C with primary antibodies against ICAM5 (1:1000, Abcam), NME4 (1:500, Santa Cruz), and GAPDH (1:5000, Proteintech). After washing, membranes were incubated with HRP-conjugated secondary antibodies (1:5000) for 1 h at room temperature. Protein bands were visualized using an ECL kit (Servicebio) and imaged with a Tanon 5200 system. Band intensities were quantified using ImageJ software, with normalization to GAPDH. All experiments were performed in triplicate.

Based on the results of the cell line experiments, total RNA was isolated from six pairs of human GC tissues and matched adjacent normal tissues using TRIzol reagent (Biomed, China) following the protocol. RNA purity was assessed by measuring A260/A280 ratios (range: 1.8–2.0) using a NanoDrop spectrophotometer (Thermo Fisher Scientific, US), and integrity was confirmed by 1% agarose gel electrophoresis. Then, equal amounts of RNA (1 g) were reverse-transcribed into complementary DNA (cDNA) using EasyScript^®^ One-Step gDNA Removal and cDNA Synthesis SuperMix (AE311, Genegen Biotechnology, China) following the manufacturer’s protocol. RT-qPCR was performed using PerfectStart^®^ Green qPCR SuperMix (AQ601, Genegen Biotechnology, China) on a CFX 96 system (Bio-Rad, US). The reaction mixture (25 L) contained 100 ng cDNA, 0.4 L each of forward and reverse primers (10 M), and 12.5 L SuperMix. The thermocycling conditions were set as follows: 95 °C for 1 min 30 s, followed by 40 cycles of denaturation for 20 s at 95 °C, annealing for 20 s at 64 °C, and extension for 30 s at 72 °C, with a final extension for 1 min 30 s at 72 °C. A melting curve was generated from 60 °C to 95 °C with increments of 0.4 °C per 15 s. This study was approved by the Chinese PLA General Hospital (Approval No. 2025-780). Primer sequences used in this study are detailed in **Supplementary Table 5**. The relative expression of target genes was normalized to GAPDH and calculated via the ΔCt method. Statistical significance was determined by single-tailed paired t-test.

## 3 Results

### 3.1 Screening of circulating proteins of GC in different ancestries

**Figure 2:**
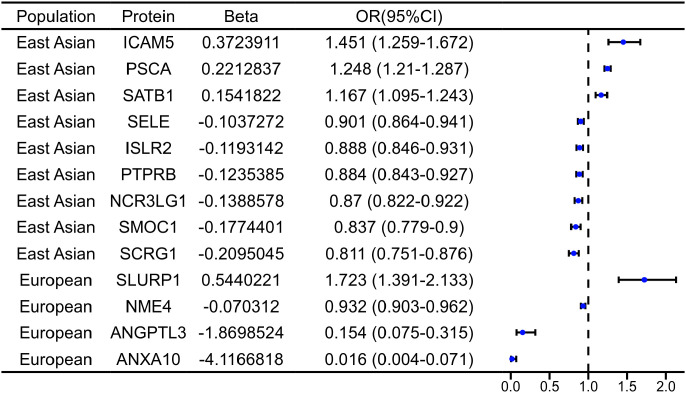
MR analysis of thirteen potential proteins in gastric cancer. CI, confidence interval; OR, odds ratio; SMR and HEIDI test verified five causal proteins.

We found that SLURP1 (odds ratio (OR) = 1.723; 95% CI: 1.391–2.133, P = 1.31E-03) exhibited a positive association with GC risk, and ANGPTL3 (OR = 0.154; 95% CI: 0.075–0.315, P = 1.42E03), NME4 (OR = 0.932; 95% CI: 0.903–0.962, P = 1.50E-02), and ANXA10 (OR = 0.016; 95% CI: 0.003–0.071, P = 1.60E-04) were negatively associated with GC risk in the EUR. In the EAS, ICAM5 (OR = 1.451; 95% CI: 1.259–1.672, P = 1.08E-03), PSCA (OR = 1.248; 95% CI: 1.210–1.287, P = 1.08E-41), and SATB1 (OR = 1.167; 95% CI: 1.095–1.243, P = 2.43E-03) were positively associated with GC risk, while SCRG1 (OR = 0.811; 95% CI: 0.751–0.876, P = 1.86E-04), PTPRB (OR = 0.884; 95% CI: 0.843–0.927, P = 5.04E-04), ISLR2 (OR = 0.888; 95% CI: 0.846–0.931, P = 1.01E-03), NCR3LG1 (OR = 0.870; 95% CI: 0.822–0.922, P = 1.13E-03), SMOC1 (OR = 0.837; 95% CI: 0.779–0.900, P = 6.97E-04), and SELE (OR = 0.901; 95% CI: 0.864–0.941, P = 1.13E-03) were negatively associated with GC risk (**Figure 2**). OR *>* 1 indicates a risk factor, while OR *<* 1 indicates a protective effect; P *<* 0.05 was considered statistically significant.The 95% CI excluding 1 indicate statistical significance at the 0.05 level. **Supplementary Table 6** provides the full results of whole-proteomic MR analysis for GC risk, and **Supplementary Table 7** provides details on the thirteen whole-proteomic MR analysis identified proteins associated with GC.

Our investigation into the potential causality and horizontal pleiotropy between significant protein expression levels and GC was conducted and revealed that ANGPTL3 successfully passed both the SMR analysis (P = 0.034) and the HEIDI test (P = 0.237) in the EUR. In the EAS, PSCA passed the SMR analysis (P = 4.75E-06).

### 3.2 Bayesian colocalization analysis supported the causality of two proteins with GC

The results of the colocalisation analysis showed that SNPs associated with GC risk *PPH*4 *>* 0.75 exist in NME4 (PPH4 = 1) (**Supplementary Figure 1**) gene expression in EUR and ICAM5 (PPH4 = 0.836) (**Supplementary Figure 2**) and SMOC1 (PPH4 = 0.770) (**Supplementary Figure 3**) gene expression in EAS, suggesting that these SNPs may mediate the effect of gene expression on GC risk. These proteins may be used as drug targets for GC. There were no common proteins in EUR and EAS. Combining this evidence, we divided the proteins into three tiers based on evidence strength. Tier 1 proteins passed three analyses (MR plus two of the three additional validations), including ANGPTL3 and ICAM5. Tier 2 proteins passed two analyses (MR plus one of the three additional validations), including NME4, SMOC1, PSCA, ANXA10 and SLURP1. Tier 3 proteins passed only MR analysis (zero additional validations), including SATB1, SCRG1, PTPRB, ISLR2, NCR3LG1, and SELE. The proteins in different ancestries are shown in **Table 1**.

**Table 1:** Mendelian randomization analysis, Summary data-based Mendelian randomization analysis and colocalization of circulating proteins in gastric cancer.

Protein	MR	SMR	HEIDI	Colocalization	Category
ANGPTL3	0.001417	0.033879	0.236947	NA	tier 1
ICAM5	0.001076	0.158638	0.450860	0.835779	tier 1
NME4	0.014962	NA	NA	1.000000	tier 2
SMOC1	0.000697	NA	NA	0.769592	tier 2
PSCA	1.08E-41	4.75E-06	0.001726	0.087174	tier 2
ANXA10	0.000160	0.923697	0.787298	0.061775	tier 2
SLURP1	0.001309	0.770317	0.310653	0.368713	tier 2
SATB1	0.002435	NA	NA	0.438909	tier 3
SCRG1	0.000186	NA	NA	0.563348	tier 3
PTPRB	0.000504	NA	NA	0.392632	tier 3
ISLR2	0.001010	NA	NA	0.096094	tier 3
NCR3LG1	0.001126	NA	NA	0.318645	tier 3
SELE	0.001126	NA	NA	0.468210	tier 3

### 3.3 The specific expression of proteins in different cell clusters

**Figure 3:**
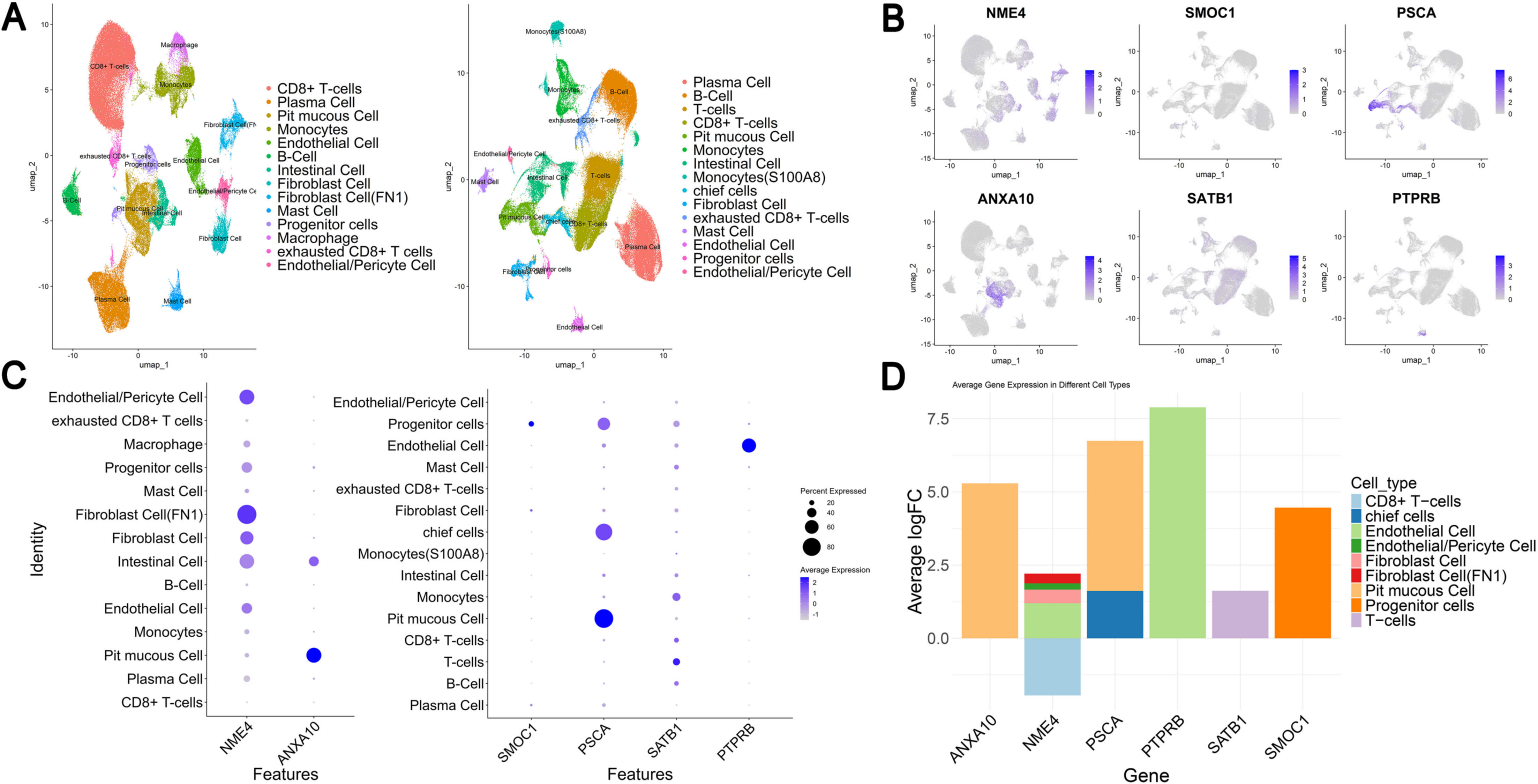
Single-cell expression of protein-coding genes in gastric cancer from European and East Asian ancestries identified by whole-proteomic Mendelian randomization. **(A)** Expression distribution of cell subpopulation clusters and cell clusters in Europeans and East Asians. **(B, C)** show the expression of protein coding genes in each cluster. **(D)** represents the different expression multiples of genes in different cell clusters.

A total of 14 cell clusters were identified in the dataset GSE183904, while 15 distinct cell clusters were identified in the dataset GSE201347 (**Figure 3A**). In the EUR cohort, NME4 exhibited differential expression in multiple cell types, including Fibroblast Cells (FN1), Endothelial/Pericyte Cells, and CD8+ T-cells (*P <* 0.05). ANXA10 showed differential expression specifically in Pit mucous cells (*P <* 0.05). In the EAS cohort, PSCA was differentially expressed in chief cells and Pit mucous cells (*P <* 0.05), while PTPRB was differentially expressed in Endothelial cells (*P <* 0.05). Furthermore, SATB1 was differentially expressed in T-cells (*P <* 0.05), and SMOC1 was differentially expressed in Progenitor cells (*P <* 0.05) (**Figures 3B, C**). The list of marker genes and differential gene expression of different cell clusters can be found in **Supplementary Table 8**. Notably, six protein coding genes (ANXA10, NME4, PSCA, PTPRB, SATB1, SMOC1) showed cell type-specific enrichment in GC with average |*log*2*FC*| *>* 1 and *FDR <* 0.05 (**Figure 3D**).

### 3.4 Result of Drug target identification and molecular docking

**Figure 4:**
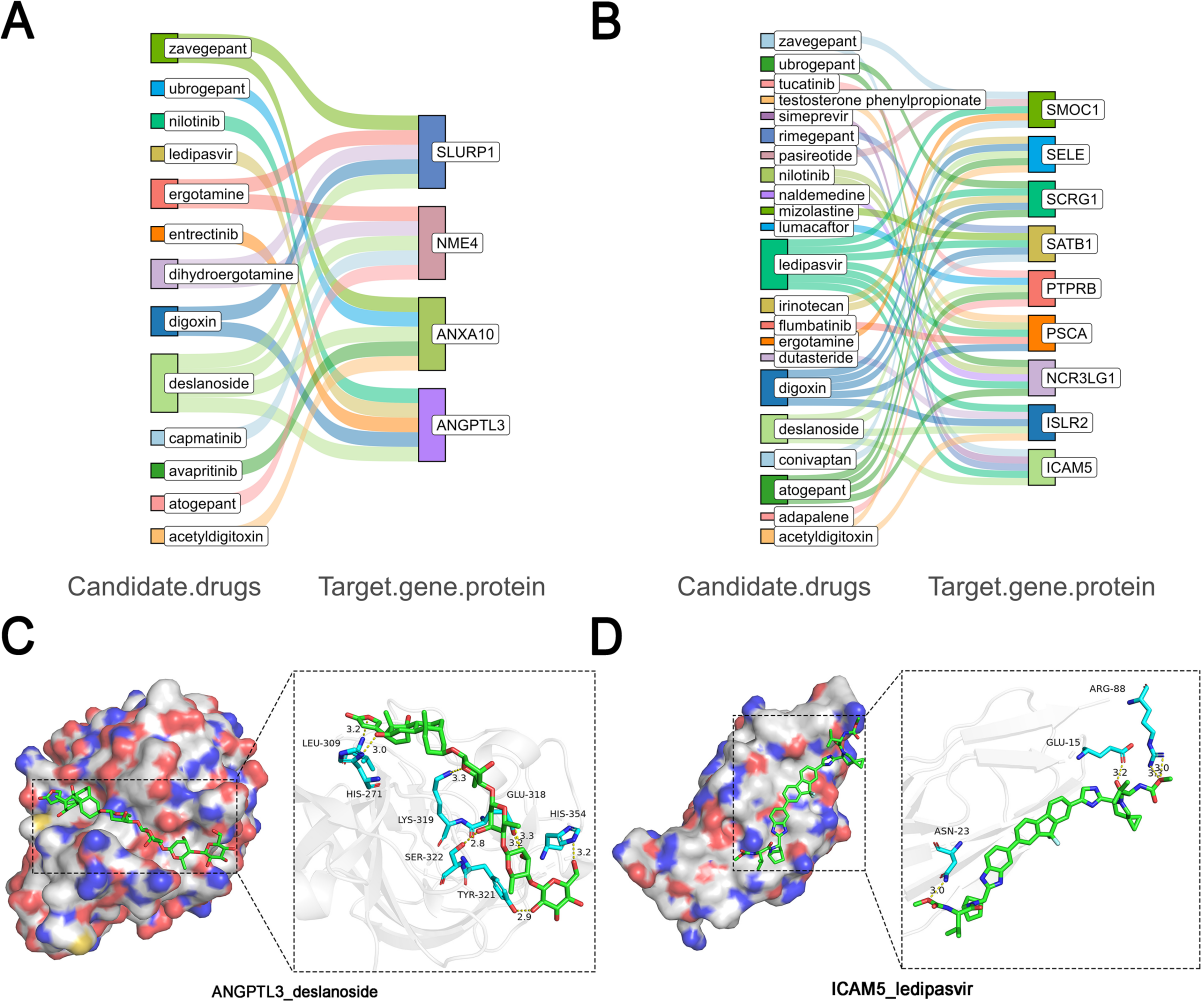
The interactions of 13 identified proteins with the top 5 molecular docking drug networks. **(A)** The result of European. **(B)** The result of East Asian. **(C)** 3D interaction patterns of ANGPTL3 and deslanoside. **(D)** 3D interaction patterns of ICAM5 and ledipasvir.

Analysis of the DrugBank and DGIdb databases identified four well-characterized drug target proteins: ANGPTL3, PSCA, PTPRB, and SELE. Among these, ANGPTL3 is clinically targeted by evinacumab, an approved therapeutic agent indicated for the treatment of hypercholesterolemia. PSCA is currently under investigation as a molecular target of MK-4721, which remains in the experimental phase of drug development. Razuprotafib has been identified as a specific inhibitor targeting PTPRB. Notably, SELE represents the most extensively targeted protein among the identified candidates, with eighteen therapeutic agents currently associated with its modulation (**Table 2**). We further performed molecular docking calculations for all potential drug target proteins with 1,622 small molecules using qvina2. The protein structure information utilized in this study is shown in **Supplementary Table 9**, and the docking binding energy results are shown in **Supplementary Table 10**. In the European cohort, deslanoside stably bound to four protein structures (*affinity <* −7.0*kcal/mol*, **Figure 4A**). Hydrogen bond interaction analysis revealed that deslanoside formed hydrogen bonds with SLURP1 (residues 70-THR, 72-SER, 80-THR, 89-HIS), NME4 (17-GLN, 54-ASP, 70-SER, 88-ARG, 113-SER, 118-HIS), ANXA10 (5-ASP, 215-GLN, 246-ARG, 248-LYS, 288-ARG), and ANGPTL3 (271-HIS, 309-LEU, 318-GLU, 319-LYS, 321-TYR, 322-SER, 354-HIS), **Figure 4C**. These results further demonstrate that deslanoside can stably interact with these four key proteins. In the EAS cohort, ledipasvir stably bound to seven core protein structures (*affinity <* −7.0*kcal/mol*, **Figure 4B**). Hydrogen bond interaction analysis showed that ledipasvir formed hydrogen bonds with ICAM5 (15-GLU, 23-ASN, 88-ARG) **Figure 4D**, ISLR2 (108-HIS, 218-GLN), NCR3LG1 (69-SER), PSCA (47-VAL, 58-SER, 60-ASN), SATB1 (406-SER, 409-LEU, 448-ARG, 523-LYS, 548-ASN), SCRG1 (39-HIS, 49-THR), and SMOC1 (105-GLN, 135-TYR, 198-ALA, 200-THR, 268-TYR). These results further confirm that ledipasvir can stably interact with these seven key proteins.

**Table 2:** Druggability for proteins that may be associated with gastric cancer risk.

Patient source	Protein	Uniprot	Drug	Drug group	Actions
European	ANGPTL3	Q9Y5C1	Evinacumab	approved, investigational	inhibitor, binder, antibody
East Asian	PSCA	O43653	MK-4721	investigational	unknown
East Asian	PTPRB	P23467	Razuprotafib	investigational	inhibitor
East Asian	SELE	P16581	Endostatin	investigational	unknown
East Asian	SELE	P16581	Carvedilol	approved, investigational	inhibitor
East Asian	SELE	P16581	Bimosiamose	investigational	inhibitor
East Asian	SELE	P16581	Uproleselan	investigational	inhibitor
East Asian	SELE	P16581	Rivipansel	investigational	inhibitor

### 3.5 Experimental validation of newly discovered drug targets

**Figure 5:**
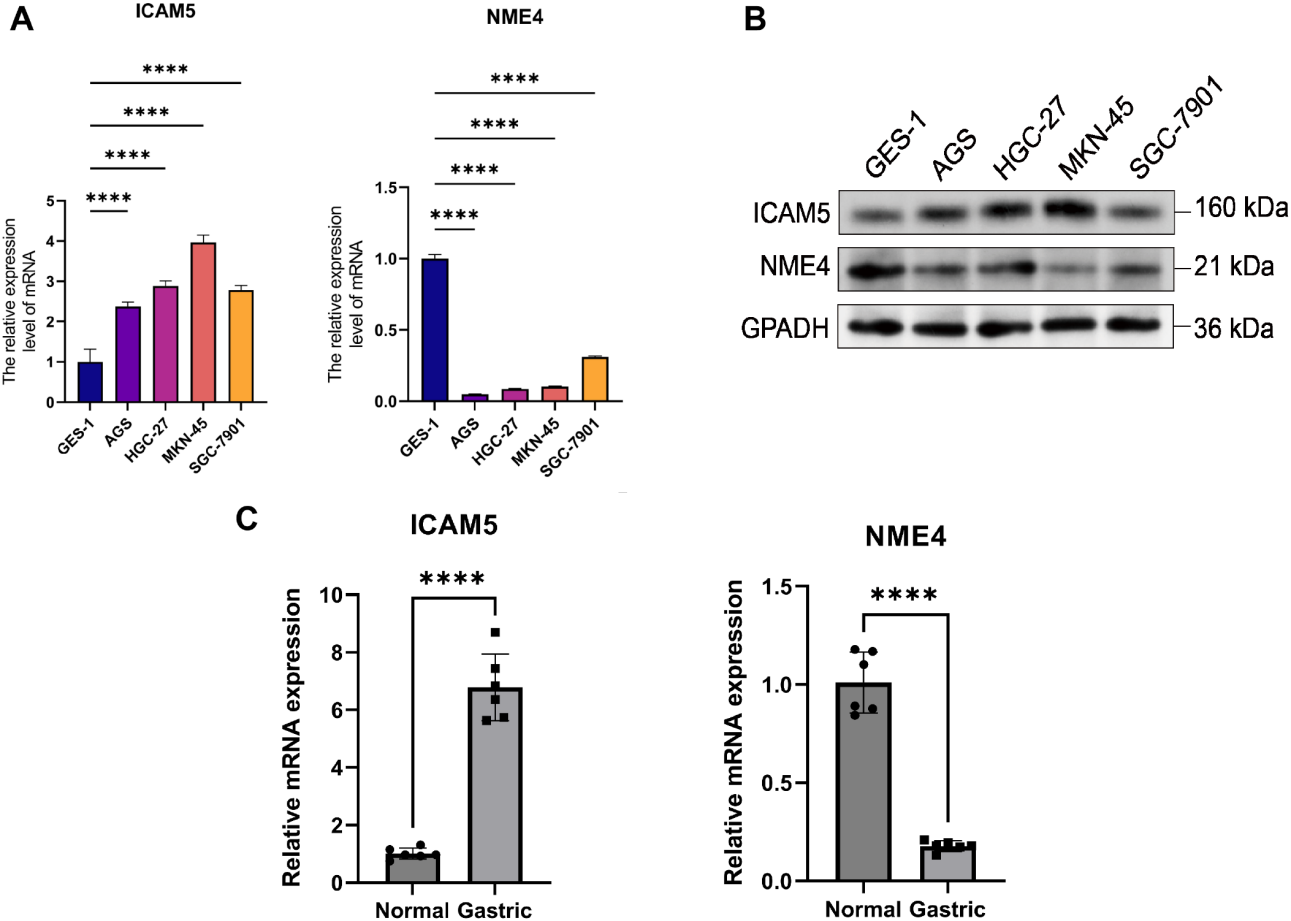
ICAM5 and NME4 expression in gastric cancer. **(A)** RT-qPCR in cell lines (AGS, HGC-27, MKN-45, SGC-7901 vs. GES-1). **(B)** Western blot in the same cell lines. **(C)** RT-qPCR in six paired cancer tissues (T) and adjacent normal tissues (N). GAPDH served as the control (**** *P* < 0.0001).

Among the 13 protein drug targets of GC that we identified through literature screening, ICAM5, NCR3LG1, NME4, PTPRB, and SMOC1, which have not been reported in the literature to have a regulatory relationship with GC, were classified as newly discovered protein drug targets, and were determined by RT-qPCR in gastric mucosal cell line (GES-1) and four GC cell lines (AGS, HGC-27, MKN-45, and SGC-7901). The results showed that there were significant differences in the expression patterns of different genes (**Figure 5A**).

Using GES-1 as a control (normalized to 1.00); the relative expression level of NME4 in GC cell lines was significantly reduced: the expression of NME4 was down-regulated by 95%, 91%, 89%, and 69% in AGS, HGC-27, MKN-45, and SGC-7901, respectively. The ANOVA results showed a highly significant difference between the groups (F = 2820, *P <* 0.0001) and an R² value of 0.9991, indicating that 99.91% of the between-group differences could be explained by changes in the relative expression of NME4. These results indicated that the relative expression levels of NME4 were significantly down-regulated in GC cells, suggesting that they may play a cancer-suppressive role in gastric carcinogenesis or development.

In contrast, compared with GES-1, the expression of ICAM5 was up-regulated by 108%, 125%, 220%, and 125% in AGS, HGC-27, MKN-45, and SGC-7901, respectively. The ANOVA results showed a highly significant difference between the groups (F = 102.9, *P <* 0.0001) and an R² value of 0.9763, suggesting that ICAM5’s relative expression changes could explain 97.63% of the between-group differences. These results indicated that the relative expression level of ICAM5 was significantly up-regulated in GC cells, suggesting that it may play a pro-carcinogenic role in gastric carcinogenesis or progression. Of note, NCR3LG1, PTPRB, and SMOC1 showed non-significant or inconsistent expression differences in some GC cell lines, which may be attributed to cell line heterogeneity or context-dependent regulation.

The WB experiments conducted on ICAM5 and NME4 revealed that the expression level of ICAM5 in GC cell lines was significantly higher than that in normal gastric mucosal epithelial cell line GES-1 (**Figure 5B**). The gray value analysis indicated that compared with GES-1, the relative expression levels of ICAM5 in AGS, HGC-27, MKN-45, and SGC-7901 increased by 23%, 58%, 58%, and 32% respectively, and the variance analysis showed that the differences between groups were significant (F = 10.21, R² = 0.8034). The expression level of NME4 in GC cell lines was significantly lower than that in normal gastric mucosal epithelial cell line GES-1 (*p <* 0.0001). The gray value analysis indicated that compared with GES-1, the relative expression levels of NME4 in AGS, HGC-27, MKN-45, and SGC-7901 decreased by 45%, 47%, 73%, and 45% respectively, and the variance analysis showed that the differences between groups were extremely significant (F = 23.61, R² = 0.9043).The experiments conducted in GC tissues yielded the same expression trend (**Figure 5C**).

## 4 Discussion

GC is highly malignant and characterized by poor prognosis, underscoring the critical need for identifying novel drug targets. In this study, we conducted a multi-ancestry proteome-wide MR analysis to identify potential drug targets for GC. The main novelty of our study lies in the discovery of five previously unreported candidate proteins associated with GC risk—ICAM5, NME4, PTPRB, SMOC1, and NCR3LG1.The identification of cross-ancestry drug candidates such as digoxin. Importantly, by comparing EUR and EAS populations, we revealed ancestry-specific target profiles, highlighting the necessity of diverse population sampling in drug target discovery.

Among the novel targets, ICAM5 exhibited the strongest evidence (Tier 1) and represents the most promising candidate for further investigation. ICAM5, a member of the intercellular adhesion molecule family, has been implicated in several cancers through large-scale association studies (11, 35, 36), but its role in GC has not been previously established. Our study provides the first causal evidence linking elevated ICAM5 protein levels to increased GC risk. Mechanistically, ICAM5 may activate the MAPK/ERK signaling pathway (37), a key driver of GC development, and inhibition of ERK has been shown to attenuate its oncogenic effects (38). Clinically, ICAM5 could serve as a potential diagnostic biomarker or therapeutic target for GC. Future studies should explore the feasibility of targeting ICAM5 using monoclonal antibodies or small molecule inhibitors.

NME4 showed a protective effect against GC (OR = 0.932). Our experimental validation confirmed that NME4 expression was significantly downregulated in both GC cell lines and clinical tissues, suggesting a tumor-suppressive role. Although NME family members have been implicated in cancer metastasis, the specific role of NME4 in GC has not been previously reported. Restoring NME4 expression or enhancing its activity may represent a novel therapeutic strategy for GC.

Other novel candidates, including PTPRB,SMOC1 and NCR3LG1 warrant further investigation. Although they did not meet the Tier 1 criteria due to lack of SMR or colocalization support, their consistent association with GC risk in MR analysis makes them interesting candidates for future functional studies.

Our analysis also successfully identified several previously reported GC-associated targets, including ANGPTL3, PSCA, ANXA10, SATB1, SLURP1, SCRG1,ISLR2 and SELE. Among these, ANGPTL3 exhibited the most compelling evidence (Tier 1) in the EUR population. Consistent with previous studies showing that ANGPTL3 inhibits GC cell proliferation and metastasis (39, 40), our MR analysis confirmed its protective effect. The DrugBank database identifies ANGPTL3 as the target of evinacumab, an approved drug for hypercholesterolemia (41). Given the inverse association between ANGPTL3 and GC risk (42), evinacumab may hold promise as a repurposed therapeutic agent for GC. Similarly, PSCA has been reported as an independent prognostic factor in GC (43). We identified PSCA as a target of MK-4721, a monoclonal antibody that has completed clinical trials for pancreatic and prostate cancer (30), suggesting its potential for GC treatment. Single-cell RNA sequencing revealed that SATB1 was differentially expressed in T-cells.Given this finding, SATB1, a genome organizer critical for T-cell development and function, has been reported to enhance regulatory T-cell activity and promote tumor immune evasion in the tumor microenvironment (44). Therefore, its differential expression in T-cells suggests a protumor effect in GC, supporting SATB1 as a potential immunotherapeutic target (45). Other known targets, including ANXA10 (46), SLURP1 (47), SCRG1 (48), and SELE (49), have been previously associated with GC prognosis or progression, and our findings further support their relevance as potential drug targets.

The molecular docking analysis revealed several candidate drugs with high binding affinity to the identified targets. In the EUR ancestry, deslanoside—a compound with known anticancer properties (50, 51)—showed strong binding to multiple targets. In the EAS ancestry, ledipasvir, an antiviral agent with demonstrated anti-cancer properties (52), exhibited high binding affinity to most identified targets. Notably, digoxin emerged as a common candidate across both ancestries. By inhibiting Na+/K+-ATPase and activating the Na+/Ca2+ exchanger, digoxin may exert anti-GC effects through the peroxisome proliferator-activated receptor and apoptotic caspase cascade pathways (53). These findings highlight the potential of drug repurposing for GC treatment and warrant further pharmacological validation.

This study has several notable strengths. First, by integrating pQTL data from DeCODE and UKBPPP with five independent GC GWAS datasets across EUR and EAS ancestries, we performed a systematic multi-ancestry drug target screening, addressing the prevailing EUR-centric bias in the field. Second, we established a multi-layered evidence framework combining MR, SMR/HEIDI, colocalization, scRNA-seq, molecular docking, and experimental validation, enabling more rigorous causal inference than singlemethod approaches. Third, we provided experimental validation for ICAM5 and NME4 in GC cell lines and clinical tissues, representing a significant original contribution to GC drug target discovery.

Several limitations should be acknowledged. First, the EAS population pQTL data had a limited sample size, necessitating a relaxed IV selection threshold (*P <* 5 × 10^−6^). However, multiple compensatory measures were implemented to mitigate false positives. Second, our analysis is predominantly based on whole blood data, which may not accurately reflect protein levels in target tissues. Third, despite pleiotropy correction, residual horizontal pleiotropy cannot be entirely excluded. Fourth, although we validated ICAM5 and NME4 expression by RT-qPCR and Western blotting, their functional roles require further investigation via gain and loss of function assays. Fifth, the anti-cancer efficacy of candidate drugs (e.g., digoxin, deslanoside, ledipasvir) was not evaluated in cellular or animal models. Future studies will focus on functional characterization of these targets and systematic evaluation of the candidate drugs using *in vitro* and *in vivo* models.

## 5 Conclusions

Our study identified five novel candidate drug targets for GC (ICAM5, NME4, PTPRB, SMOC1, and NCR3LG1), with ICAM5 and NME4 showing the strongest evidence and experimental validation. We also demonstrated ancestry-specific target profiles, highlighting the importance of diverse population sampling in drug discovery. Evinacumab, MK-4721, deslanoside, digoxin, and ledipasvir represent potential repurposed drugs for GC treatment. These findings provide valuable evidence for the development of multi-ancestry drug targets for GC.

## Data Availability

The datasets presented in this study can be found in online repositories. The names of the repository/repositories and accession number(s) can be found in the article/**Supplementary Material**.

## Supplementary material

Supplementary Table 1 Data source.

Supplementary Table 2 European pqtl.

Supplementary Table 3 East Asian pqtl.

Supplementary Table 4 DECODE pqtl.

Supplementary Table 5 Primer sequences used in this study.

Supplementary Table 6 All results of discovery proteome-wide Mendelian randomization for GC risk.

Supplementary Table 7 Results of 13 proteome-wide Mendelian randomization identified proteins for GC.

Supplementary Table 8 seRNA-seq result.

Supplementary Table 9 DGIdb result.

Supplementary Table 10 Protein structure information.

Supplementary Table 11 Molecular docking result.

Supplementary Figure 1 NME4 locuscomparer.

Supplementary Figure 2 ICAM5 locuscomparer.

Supplementary Figure 3 SMOC1 locuscomparer.

Supplementary File Molecular docking visualisation.

## Glossary

GC: Gastric cancer
Pqtl: Protein quantitative trait loci
EUR: European
EAS: East Asian
GWAS: Genome-wide association studies
SMR: Summary data-based MR
scRNA-seq: Single-cell sequencing
IVs: Instrumental variables
IVW: Includinginverse-variance weighted
DEG: Differentially expressed genes
PCA: Principal component analysis
ICAM5: Intercellular Adhesion Molecule 5
SMOC1: SPARC Related Modular Calcium Binding 1
PSCA: Prostate Stem Cell Antigen
SATB1: SATB Homeobox 1
SCRG1: Stimulator Of Chondrogenesis 1
PTPRB: Protein Tyrosine Phosphatase Receptor Type B
ISLR2: Immunoglobulin Superfamily Containing Leucine Rich Repeat 2
NCR3LG1: Natural Killer Cell Cytotoxicity Receptor 3 Ligand 1
SELE: Selectin E
ANGPTL3: Angiopoietin Like 3
NME4: Nucleoside Diphosphate Kinase 4
ANXA10: Annexin A10
SLURP1: Secreted LY6/PLAUR Domain Containing 1
