# Supplementary figures and images for: Plasma proteome-wide Mendelian randomization reveals multi-ancestry drug targets for gastric cancer
Source: Front Oncol. 2026 May 1;16:1821512. doi: 10.3389/fonc.2026.1821512 (PMC13175846; doi:10.3389/fonc.2026.1821512)

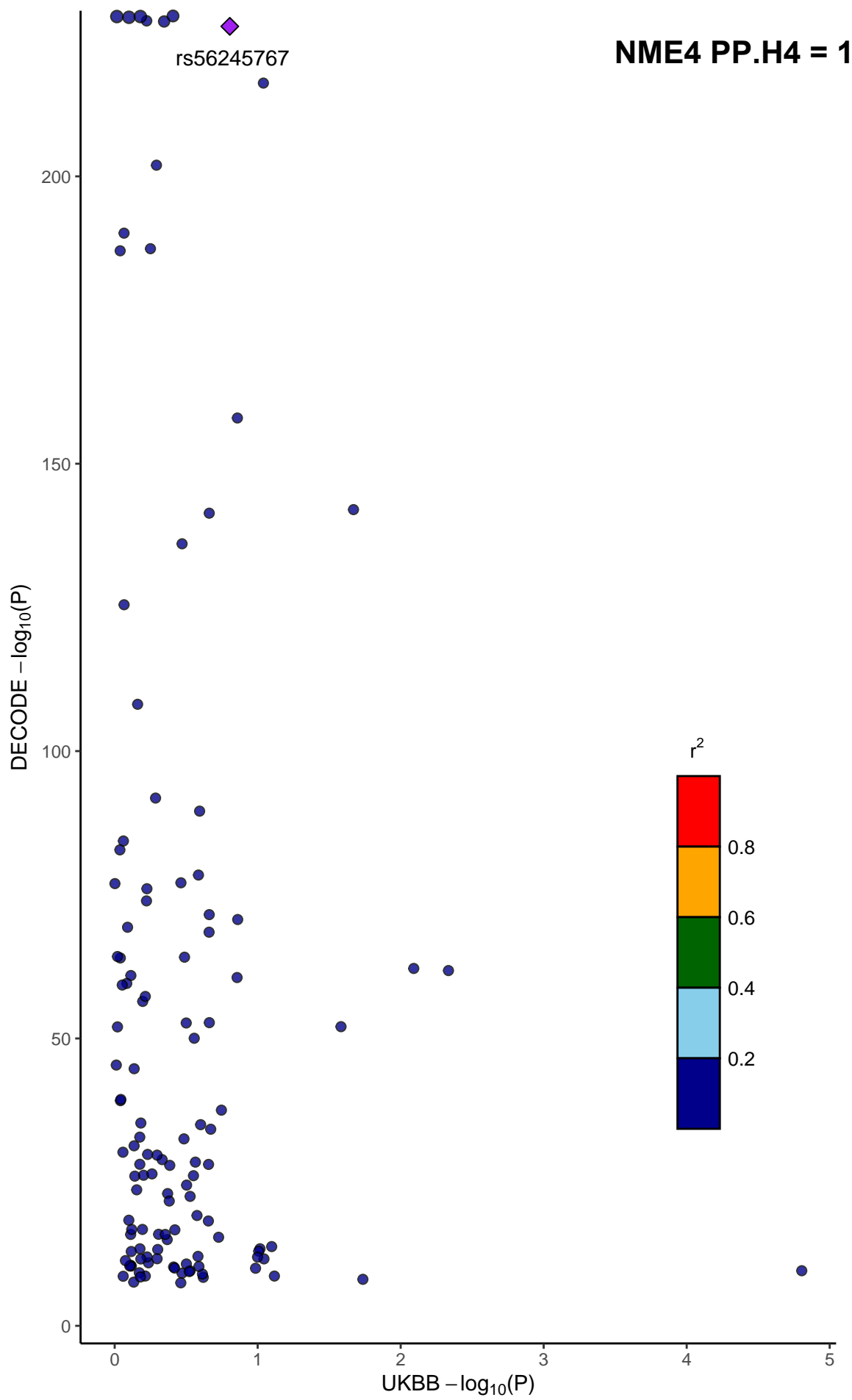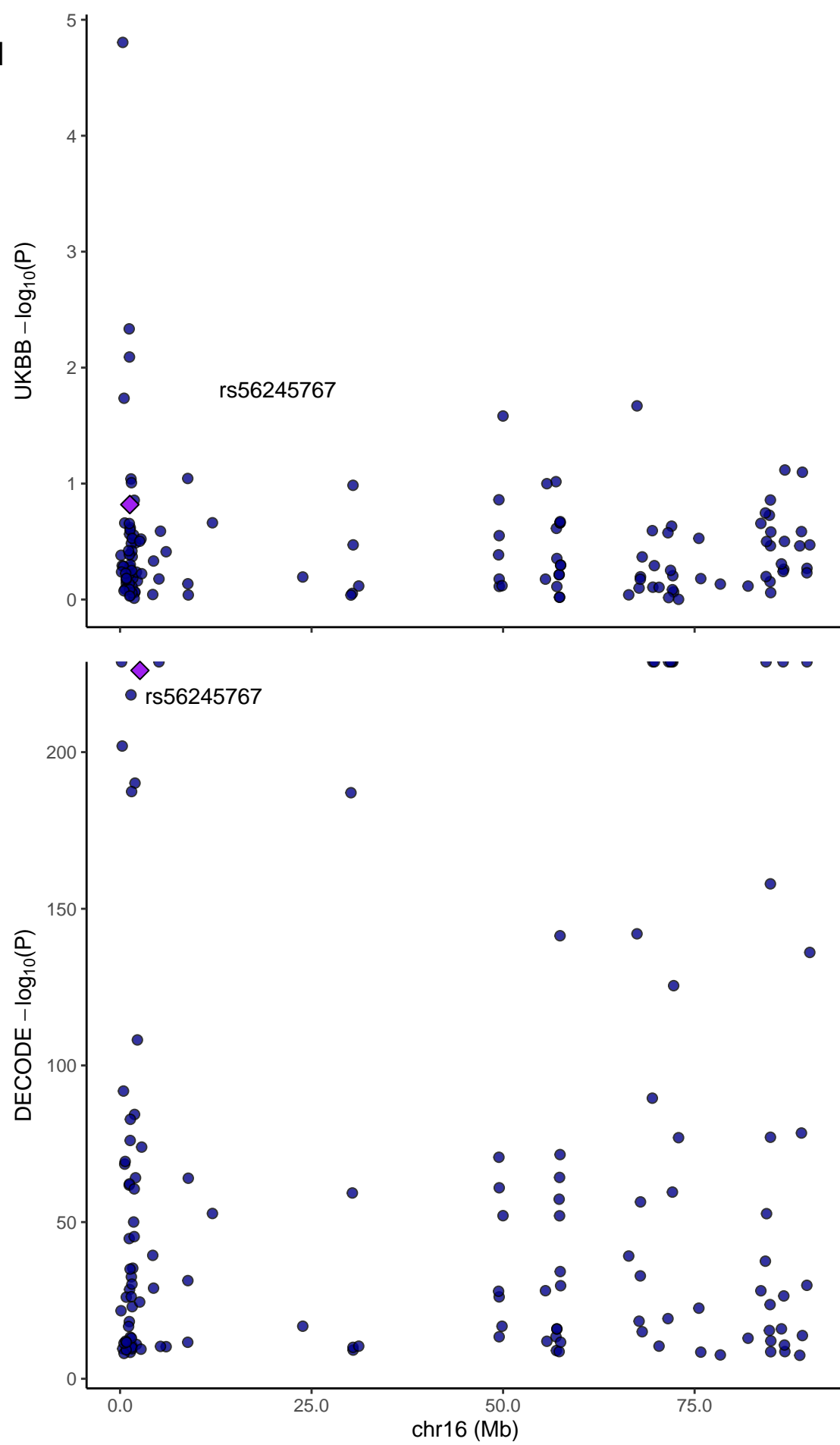

Supplement: Supplementary Figure 1 — NME4 locuscomparer. [file Image1.pdf]

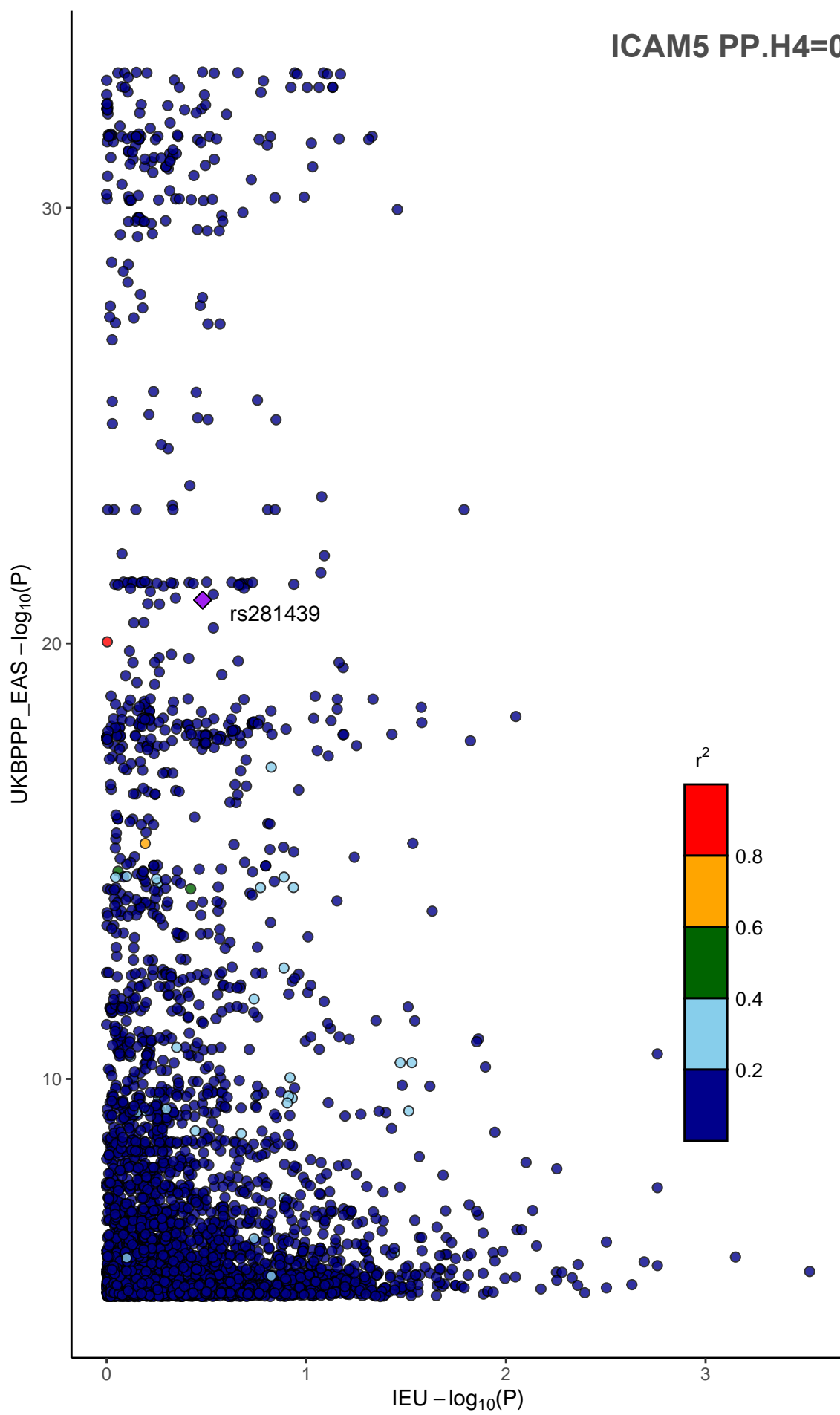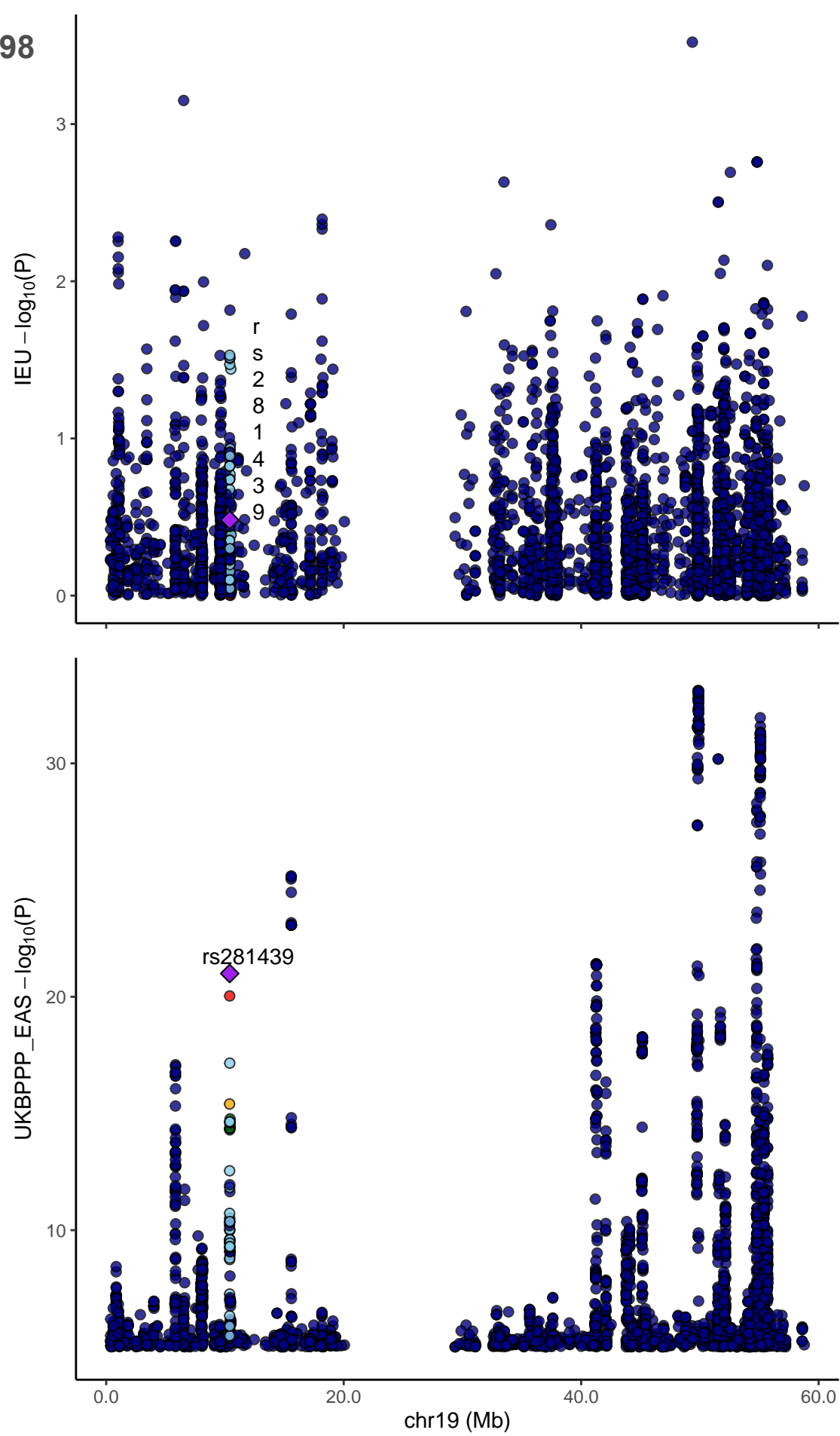

Supplement: Supplementary Figure 2 — ICAM5 locuscomparer. [file Image2.pdf]

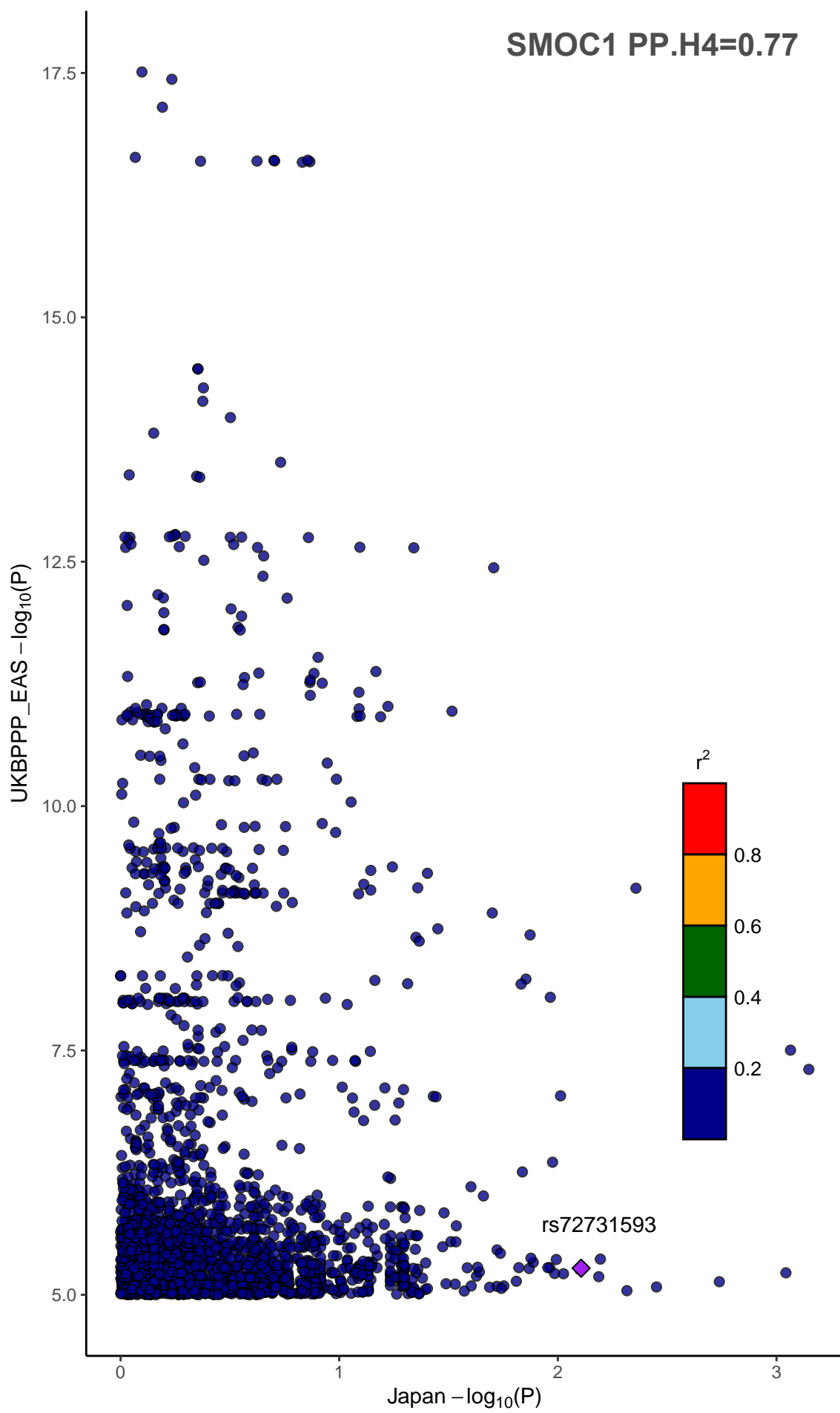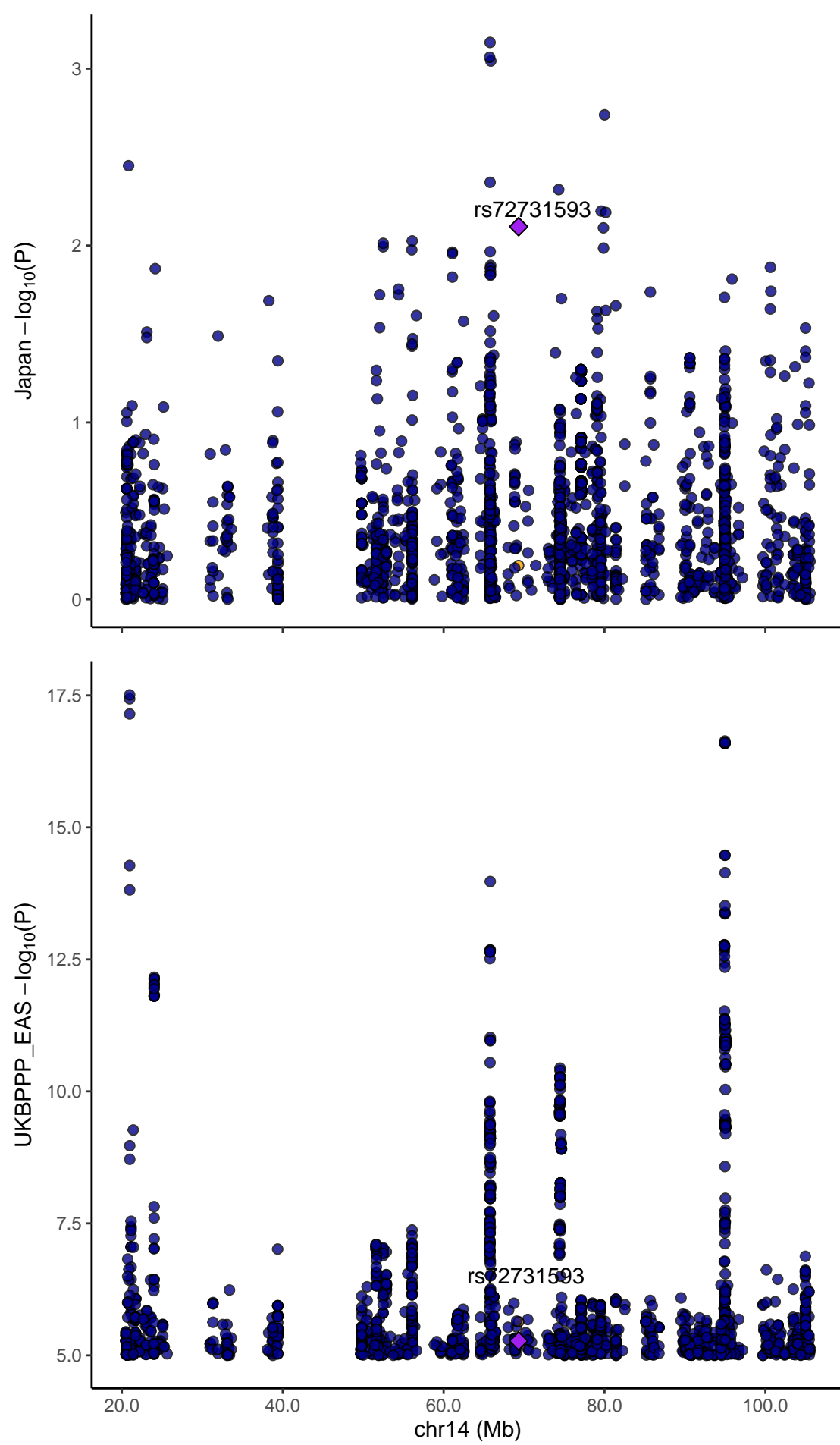

Supplement: Supplementary Figure 3 — SMOC1 locuscomparer. [file Image3.pdf]
