# Supplementary material for: Plasma proteome-wide Mendelian randomization reveals multi-ancestry drug targets for gastric cancer
Source: Front Oncol. 2026 May 1;16:1821512. doi: 10.3389/fonc.2026.1821512 (PMC13175846; doi:10.3389/fonc.2026.1821512)
Supplement: Supplementary File — Molecular docking visualisation. [file SupplementaryFile1.pdf]

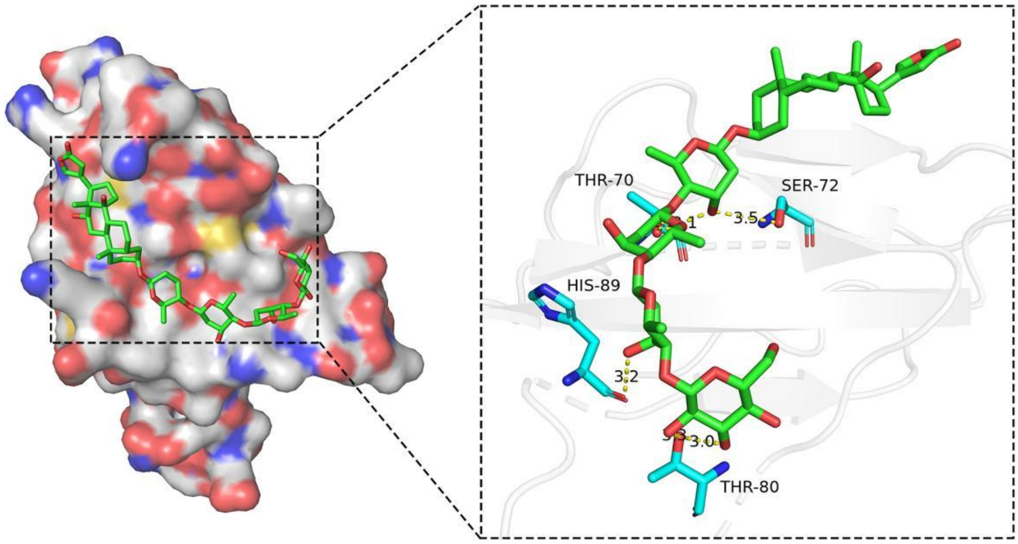

SLURP1_deslanoside

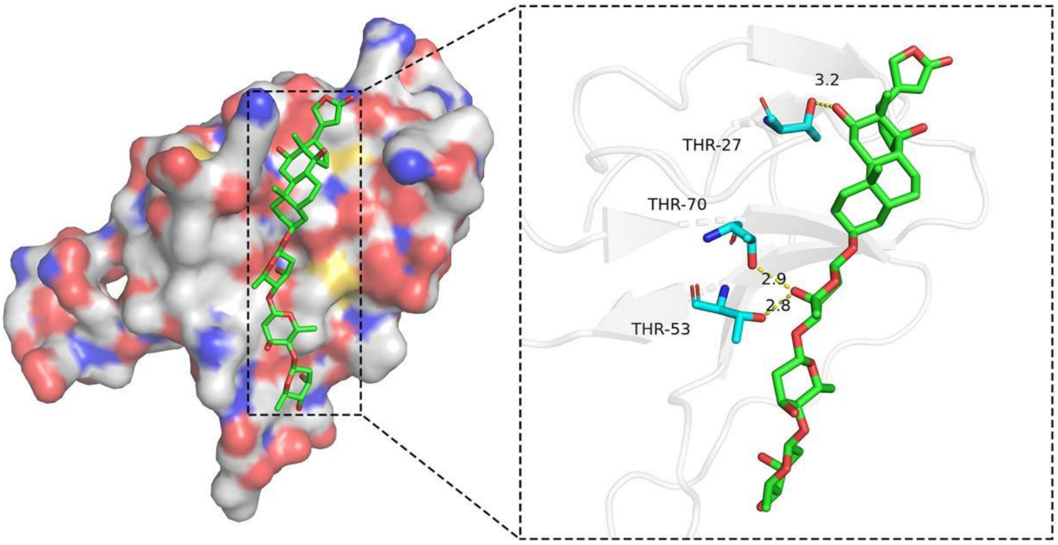

SLURP1_digoxin

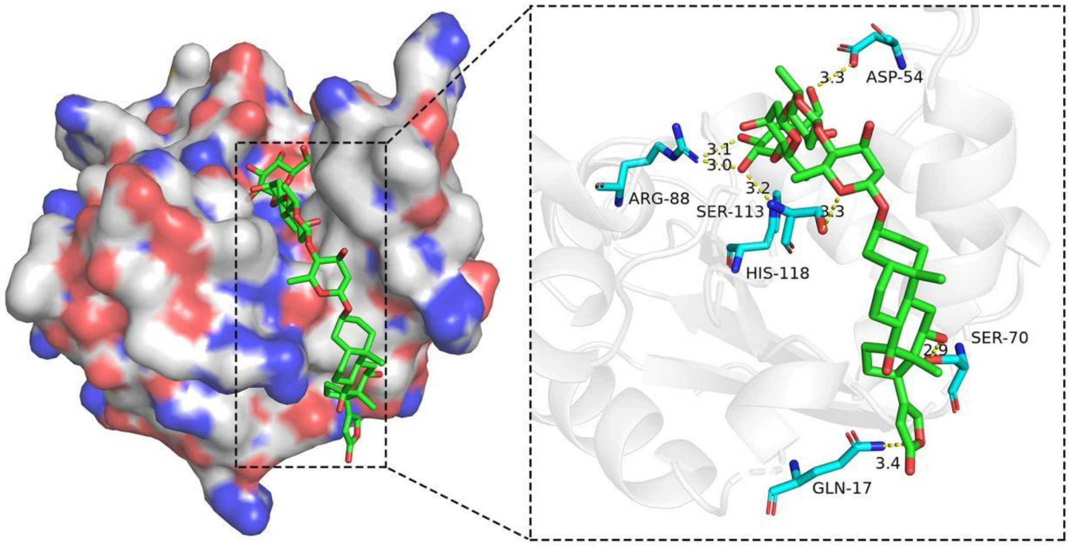

NME4_deslanoside

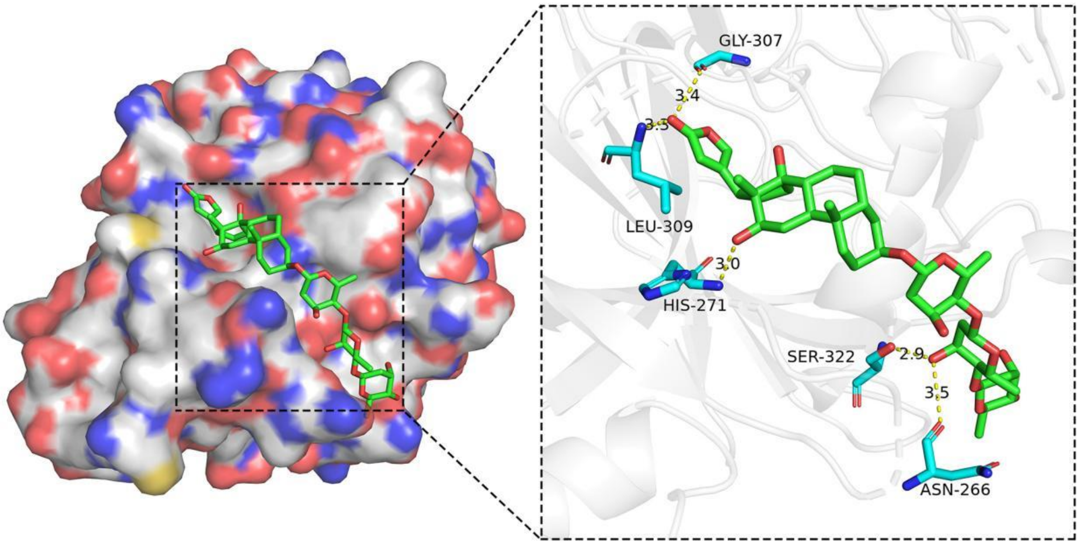

ANGPTL3_digoxin

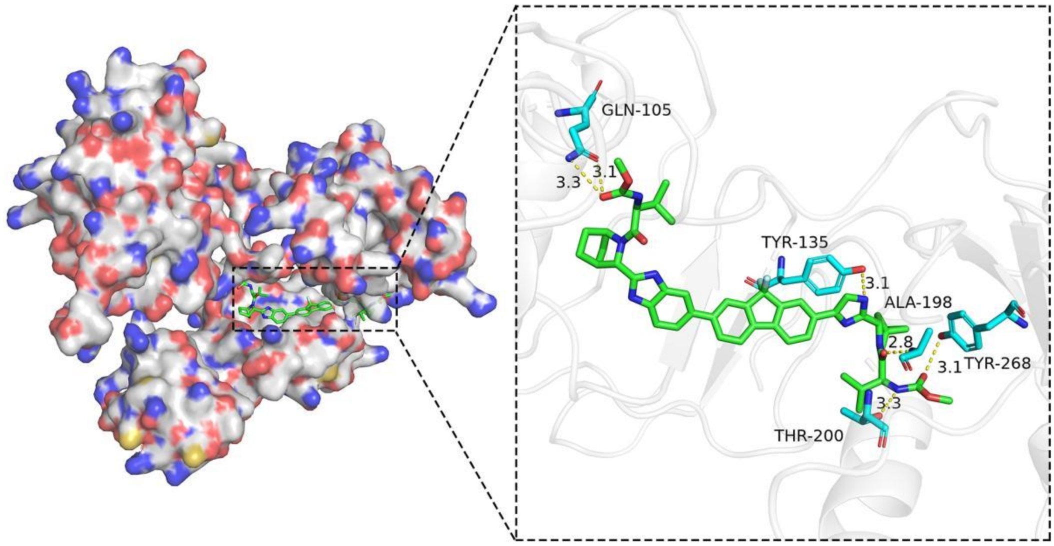

SMOC1_ledipasvir

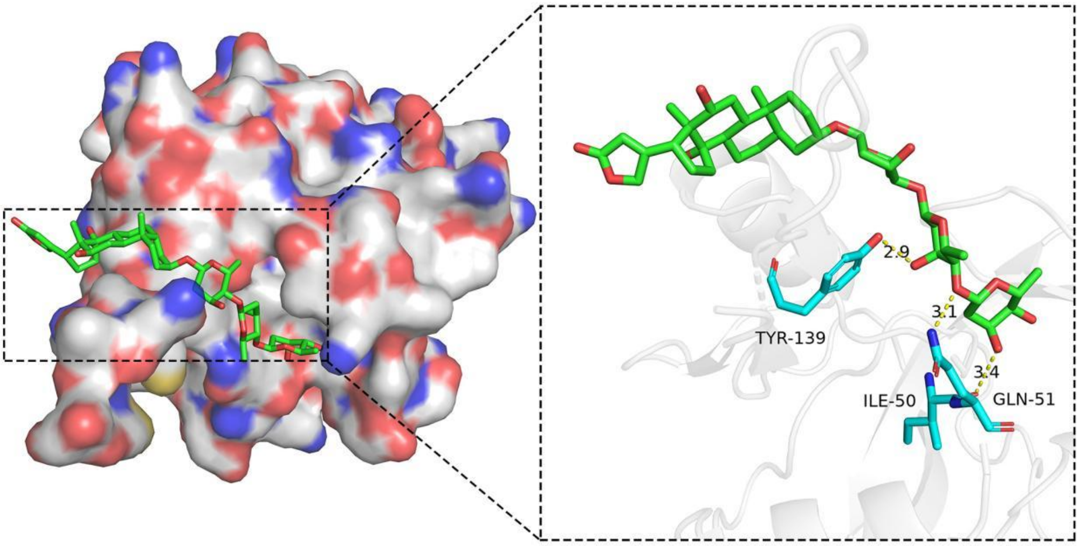

SELE_digoxin

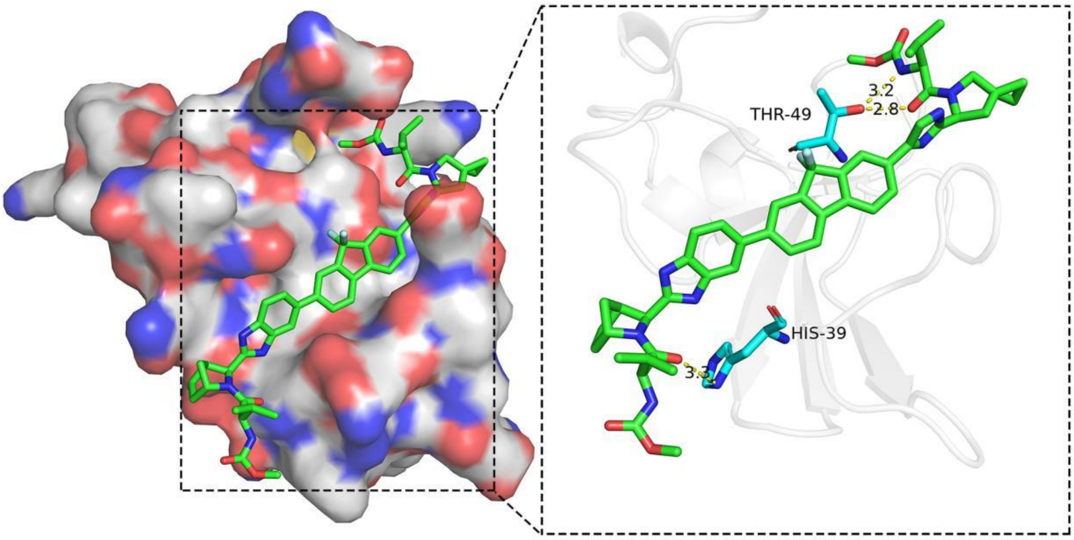

SCRG1_ledipasvir

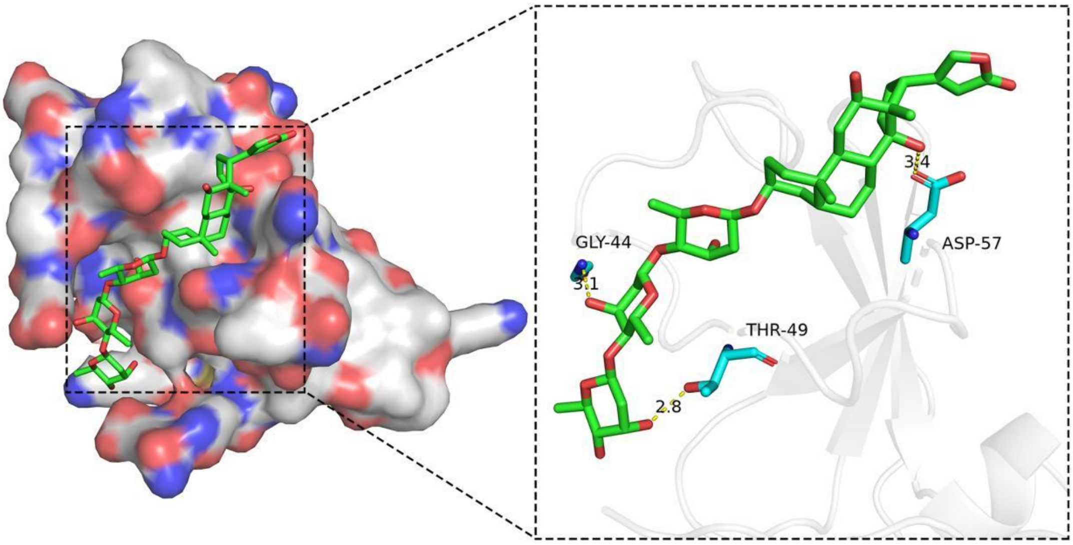

SCPG1_digoxin

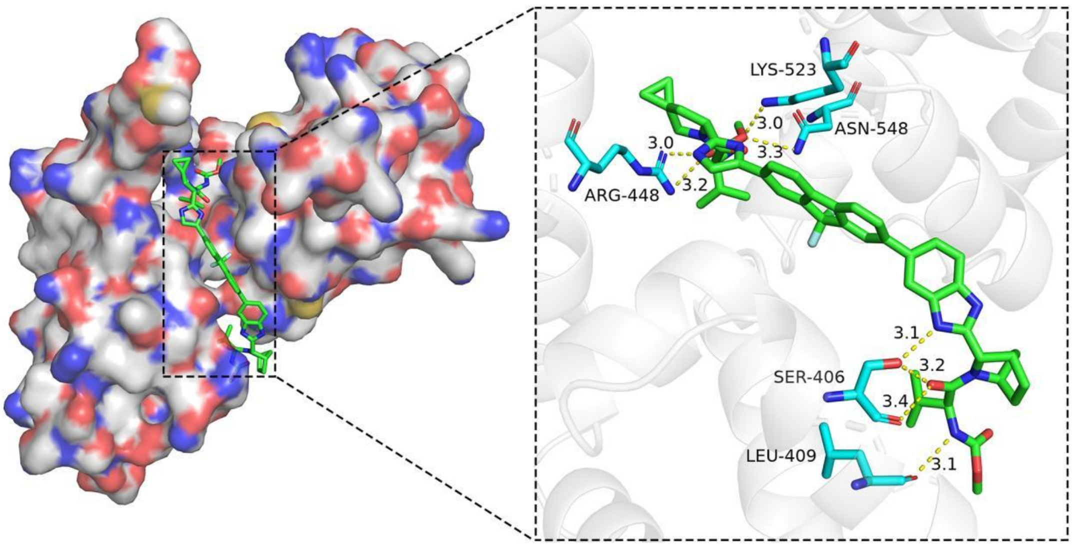

SATB1_ledipasvir

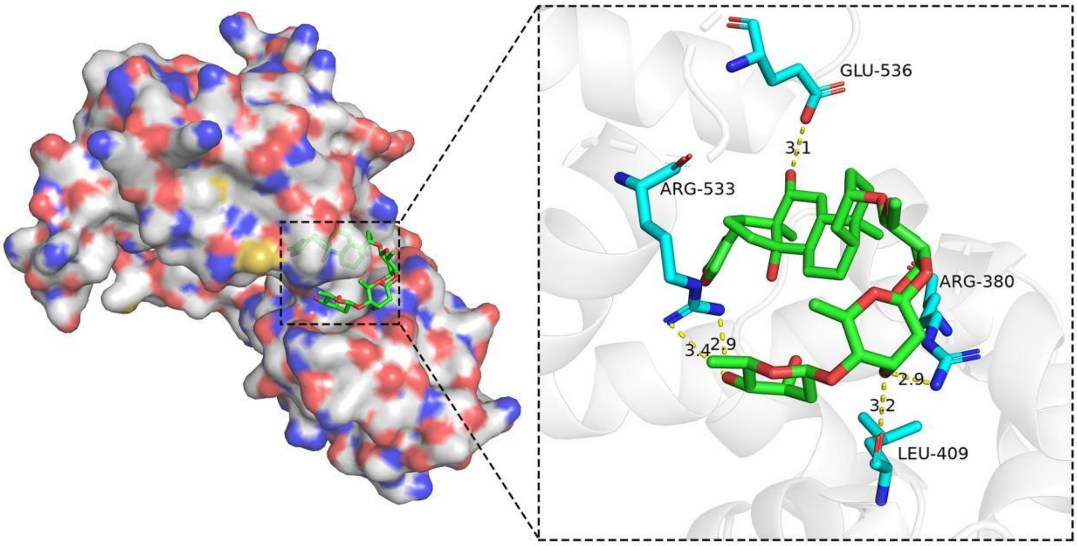

SATB1_digoxin

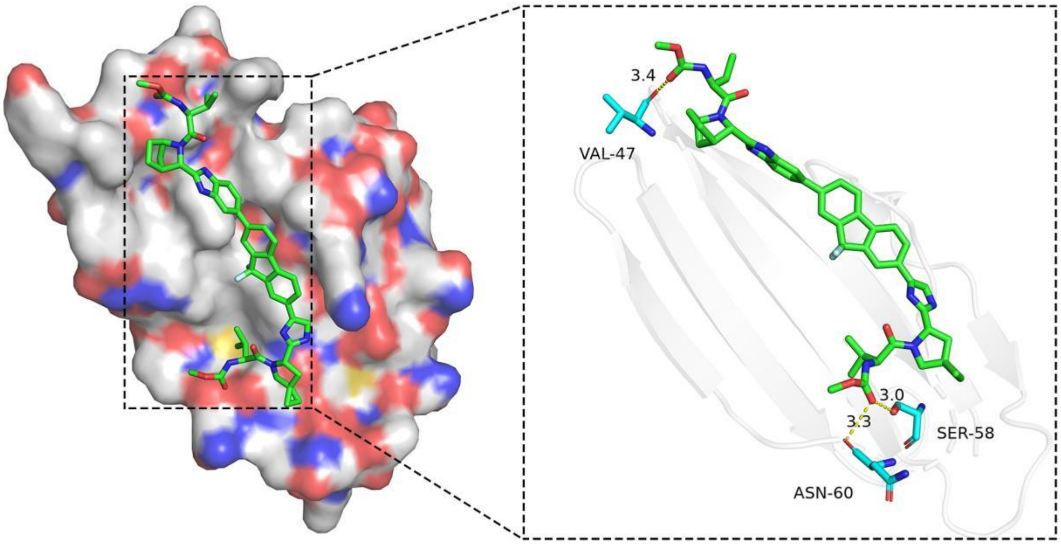

PSCA_ledipasvir

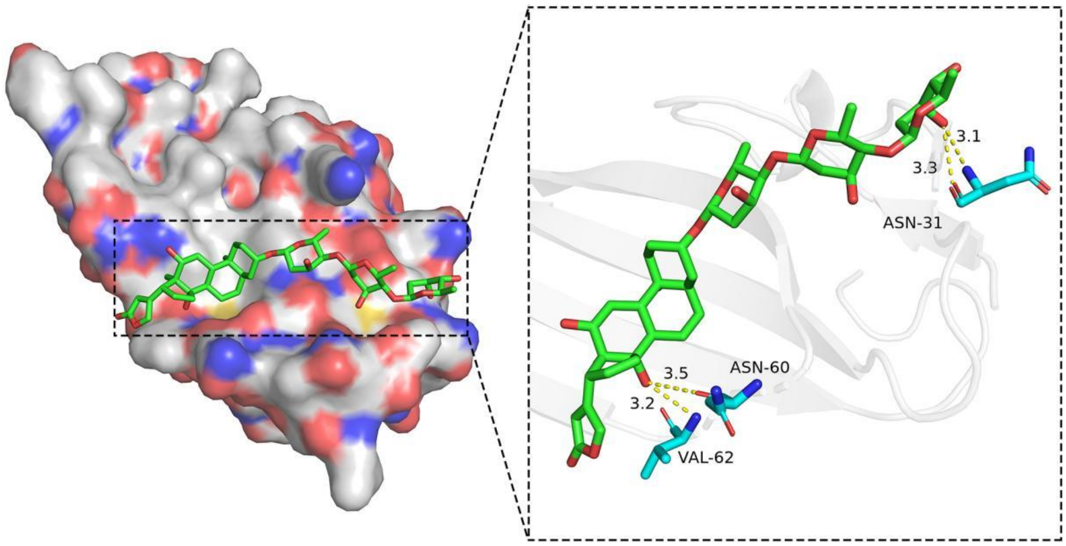

PSCA_digoxin

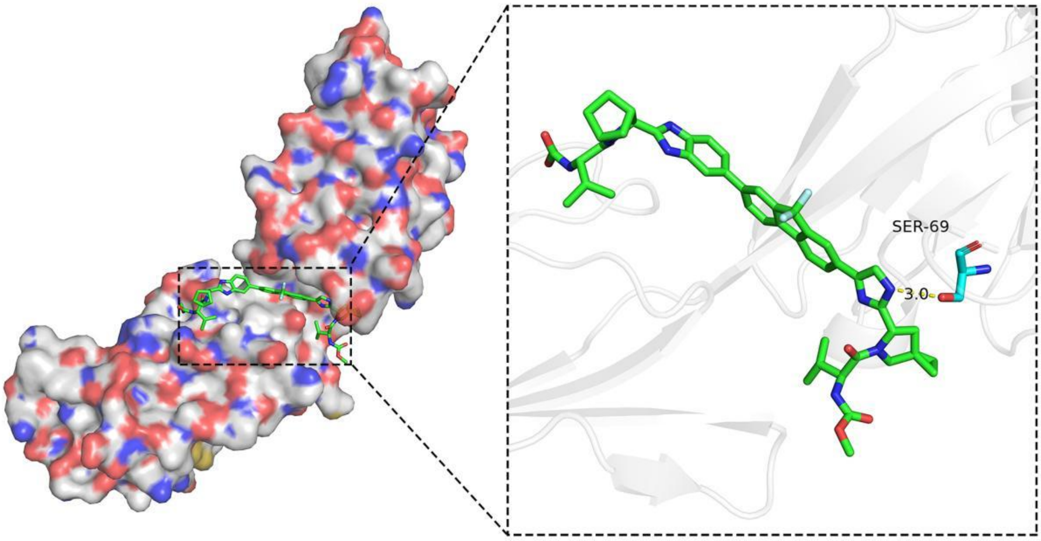

NCR3LG1_ledipasvir

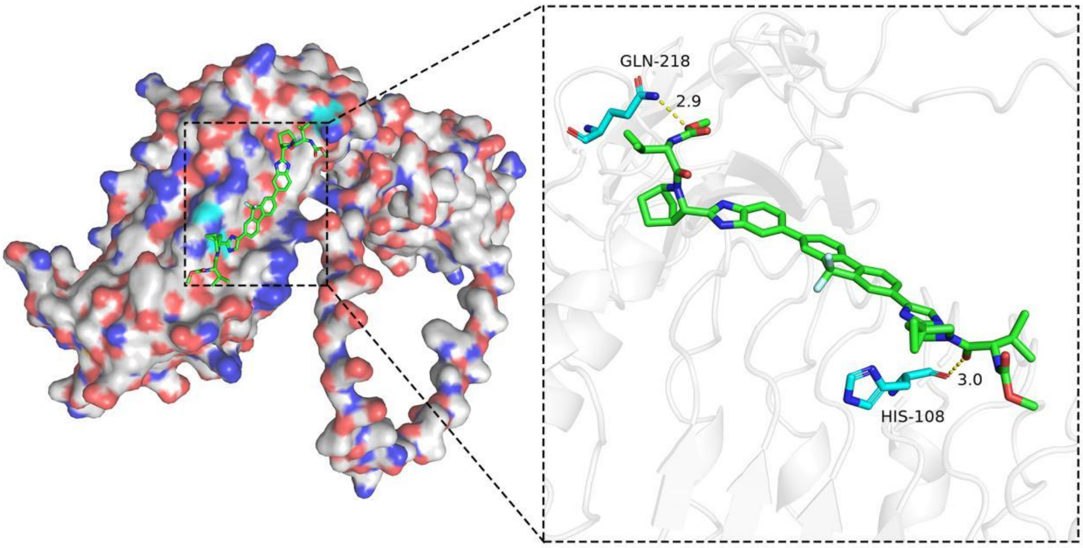

ISLR2_ledipasvir

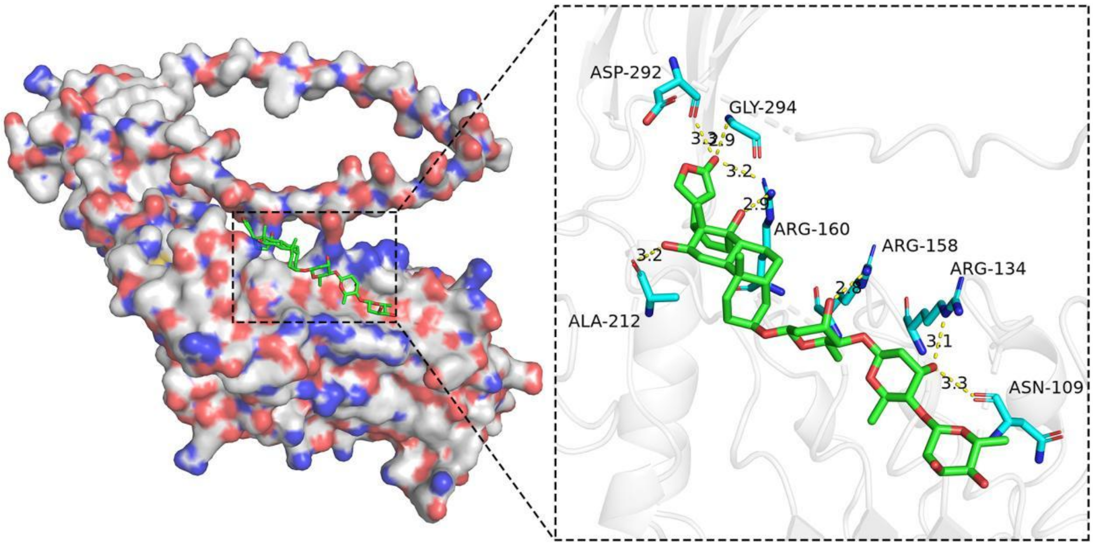

ISLR2_digoxin
